# Comparison of Vertical Jump Force–Time Metrics Between ACL-Injured and Healthy Semi-Professional Male and Female Soccer Players

**DOI:** 10.3390/sports12120339

**Published:** 2024-12-06

**Authors:** Dimitrije Cabarkapa, Damjana V. Cabarkapa, Andrew C. Fry, Yu Song, Thordis Gisladottir, Milos Petrovic

**Affiliations:** 1 Jayhawk Athletic Performance Laboratory—Wu Tsai Human Performance Alliance, Department of Health, Sport and Exercise Sciences, University of Kansas, Lawrence, KS 66045, USA; 2 Serbian Institute of Sport and Sports Medicine, 11000 Belgrade, Serbia; 3 Biomechanics Laboratory, Department of Health, Sport and Exercise Sciences, University of Kansas, Lawrence, KS 66045, USA; 4 Research Center for Sport and Health Sciences, University of Iceland, 102 Reykjavik, Iceland

**Keywords:** injury, recovery, test, monitoring, rehabilitation, force, power, eccentric, concentric

## Abstract

Given the increasing use of innovative force plate systems in applied sports settings and the impact that anterior cruciate ligament (ACL) injuries have on team success, the purpose of the present study was to compare the lower-body neuromuscular performance characteristics of athletes who underwent ACL reconstruction (ACLR) and their non-injured counterparts (i.e., healthy controls). Forty-five male (thirteen injured) and twenty-six female (ten injured) semi-professional soccer players volunteered to participate in the present study. Each athlete performed three countermovement vertical jumps (CMJs) while standing on a uniaxial force plate system sampling at 1000 Hz. The injured athletes completed a nine-month recovery protocol and were screened 11–13 months post-ACLR. The dependent variables included the force–time metrics within both the eccentric and concentric phases of the CMJ. Independent *t*-tests or Mann–Whitney U-test were used to examine statistically significant (*p* < 0.05) differences in each variable (i.e., ACL-injured vs. healthy controls). The results revealed no significant between-group differences in any CMJ force–time metrics of interest (e.g., concentric peak force, eccentric mean power, countermovement depth) between ACL-injured and non-injured athletes, including inter-limb asymmetry measures (i.e., peak takeoff and landing force). Besides implying the effectiveness of the implemented rehabilitation protocol, these findings suggest that the CMJ may not present a sufficient neuromuscular performance stimulus needed to expose lower-limb asymmetries and strength and power deficiencies 11–13 months post-ACLR.

## 1. Introduction

Soccer is one of the most popular international sports, with approximately 265 million participants worldwide [1,2]. It is a team sport that requires athletes to perform repetitive high-intensity actions such as rapid accelerations, decelerations, jumping, and sudden change of direction movements, regardless of the playing position, skill level, and tactical strategies implemented by the coaching staff [3,4]. The aforementioned sport-specific demands that soccer athletes are exposed to during practice and competition place them at a considerably greater incidence of anterior cruciate ligament (ACL) injury when compared to the general population [2,5]. In certain instances, the likelihood of injury occurrence may be twice as often among athletes than people injured in non-athletic accidents [6]. In addition, ACL reconstruction (ACLR) in the nondominant limb was found to be directly related to an increased risk of another ACL injury in the dominant limb, especially in female soccer players [7]. Moreover, ACL injury occurrence tends to be notably greater for female than male athletes due to hormonal status, ligament size, quadriceps angle, and tibial slope [8]. Thus, preventive screening and evaluation procedures implemented by sports practitioners can be critical in identifying athletes at increased risk of ACL injury and decreasing the likelihood of injury occurrence [9].

Jump-landing tasks have often been utilized to obtain a deeper insight into lower-body biomechanical characteristics that may predispose athletes to ACL injury as well as track athletes’ recovery progress post-ACLR [9,10,11,12]. Some of these tests include the bilateral countermovement vertical jump (CMJ), single-leg jump, drop jump, or their combinations (e.g., single-leg drop jump), performed on laboratory-based or innovative portable force plate systems [10,11,12,13,14]. For example, a recently published study conducted on 322 junior and senior Australian football and soccer players revealed that CMJ peak takeoff force, dynamic knee valgus, and ACL injury history were capable of correctly classifying injured from non-injured athletes in 78% of cases [13]. On the other hand, Lem et al. [15] found that a deficit in single-leg drop jump height, reactive strength index (RSI), and mean and peak concentric force production persisted in the injured leg of collegiate athletes who had returned to sports 10 months post-ACLR. Similar findings were obtained by Gustavsson et al. [16], who found that jump height was an effective parameter in assessing restoration to normal function post-ACLR. In addition, Bakal et al. [10] found significant lower-limb kinetic asymmetries when comparing drop jump performance between individuals who underwent ACLR and the healthy controls. Specifically, participants who suffered ACL injury revealed notably greater asymmetries in eccentric mean force (8.0 vs. 3.4%), concentric peak force (11.6 vs. 4.4%), eccentric impulse (8.8 vs. 3.8%), and peak landing force (12.7 vs. 1.7%) when compared to their healthy counterparts [10].

While each of the aforementioned testing modalities has its pros and cons, there is still a lack of consistency about which test may offer the best insight into athletes’ performance capabilities, ability to screen athletes for an increased likelihood of ACL injury/reinjury occurrence, as well as the effectiveness of post-ACLR rehabilitative procedures [10,17,18,19,20]. Also, unlike conducting testing in a research laboratory, the testing procedures performed in an applied sports setting need to be time-efficient and minimally invasive so that they do not interfere with team training or competition schedules, especially at the professional level of play. Thus, bilateral CMJ has been one of the commonly performed tests in sport-specific settings. When performed on portable dual-force plate systems that offer instantaneous data analysis, the CMJ can provide a plethora of force–time metrics that depict lower-body neuromuscular performance characteristics as well as inter-limb asymmetries [21,22,23,24,25,26]. For example, a recently published study conducted on 369 female soccer players used a portable force plate system and found that athletes with a history of ACL injury tend to demonstrate inter-limb asymmetries in strength and landing kinetics months-to-years post-ACLR [13]. In addition, it should be noted that multiple research reports have testified to the solid validity and reliability of this innovative technology for the assessment of CMJ kinetic and kinematic performance parameters [27,28,29].

Therefore, considering an exponential increase in the implementation of portable force plates in applied sports settings and the burden that ACL injuries have on overall team success, the purpose of the present study was to compare the lower-body neuromuscular performance profiles of semi-professional soccer athletes who underwent ACLR (i.e., male and female) and their non-injured counterparts (i.e., healthy controls). Based on the previously published scientific literature, it is hypothesized that notable between-group differences will be observed in force–time metrics of interest, within both the eccentric and concentric phases of the CMJ.

## 2. Materials and Methods

### 2.1. Participants

Forty-five male and twenty-six female semi-professional soccer players volunteered to participate in the present study. Thirteen male (age = 27.5 ± 5.1 years; body mass = 76.6 ± 9.1 kg; height = 179.3 ± 6.5 cm) and ten female athletes (age = 22.8 ± 5.9 years; body mass = 67.4 ± 11.7 kg; height = 171.6 ± 6.6 cm) had sustained ACL injuries, while the remaining thirty-two male (age = 23.2 ± 5.0 years; body mass = 80.2 ± 5.7 kg; height = 181.7 ± 5.6 cm) and sixteen female athletes (age = 24.5 ± 3.9 years; body mass = 64.0 ± 8.7 kg; height = 169.9 ± 6.7 cm) served as a healthy control group. 

The inclusion criteria for participation in this investigation were the following: (i) male and female athletes between 18 and 35 years old, (ii) sustained a complete ACL tear, (iii) underwent ACLR, (iv) completed a nine-month rehabilitation protocol in the same clinic, and (v) assessed 11–13 months post-ACLR. Conversely, the exclusion criteria were the following: (i) sustained more than an ACL tear and (ii) had any type of previous knee surgery (e.g., meniscus or medial collateral ligament). In addition, it is important to note that the data in the present study were obtained from the same clinic, indicating that the same medical staff members completed the ACLR and rehabilitation. The testing protocol performed in the present study was approved by the University of Iceland Institutional Review Board, and all athletes signed an informed consent document.

### 2.2. Procedures

Upon arrival at the laboratory, each athlete completed a warm-up protocol consisting of a low-intensity run on a treadmill (i.e., 3–5 min) and a set of dynamic stretching exercises. The warm-up was administered by the same research assistants and was standardized across all participants. Then, each athlete stepped on a uniaxial force plate system (ForceDecks Max, VALD Performance, Brisbane, Australia) sampling at 1000 Hz and performed three CMJs with no arm swing (i.e., hands on the hips during the entire movement). Each jump was separated by a 15 s rest interval and the average value across three jump trials was used for performance analysis purposes. 

The force–time metrics examined in the present study were the following: vertical jump height (i.e., impulse–momentum calculation), countermovement depth, RSI-modified (i.e., jump height divided by contraction time), eccentric and concentric peak velocity and duration, eccentric and concentric mean and peak force and power, peak takeoff and landing force asymmetry (i.e., [(right leg − left leg)/right leg] × 100%) [14]. Considering the variations in body mass across participants, the force and power metrics were expressed in relative terms (i.e., concentric mean force divided by the athlete’s body mass). The selection of the aforementioned metrics was based on previously published research reports that demonstrated solid levels of validity and reliability [21,22,26,29,30,31]. The start of the contraction was determined when the system mass fell below the 20 N threshold, which was termed the movement onset, and ended at takeoff. Takeoff was defined as the time point at which vertical force dropped below a 20 N threshold. Also, in line with manufacturer recommendations, the eccentric phase was defined as the phase containing a negative center of mass velocity.

### 2.3. Statistical Analysis

Shapiro–Wilk tests and Q–Q plots were used to examine the assumption of normality. If the variable met the normality assumption, mean and standard deviations (x̄ ± SD) were reported, and independent *t*-tests were used to determine statistically significant differences in lower-body neuromuscular performance characteristics between ACL-injured and healthy athletes, separately for male and female participants. Conversely, if the variable violated the assumption of normality, median and interquartile range (M[IQR]) were reported, and the Mann–Whitney U-test was used to make between-group comparisons for each force–time metric of interest. The α level of *p* < 0.05 was used as a criterion for statistical significance. All statistical analysis procedures were completed in SPSS (Version 28.0; Chicago, IL, USA). 

## 3. Results

Descriptive statistics (mean and standard deviations or median and interquartile ranges) for each dependent variable can be found in Table 1. Between-group comparisons revealed no statistically significant differences (*p* > 0.05) in force–time metrics of interest examined in the present study between ACL-injured and non-injured athletes (i.e., control group), within both male and female cohorts of athletes. 

## 4. Discussion

The purpose of the present study was to examine differences in lower-body neuromuscular performance characteristics between semi-professional male and female soccer players who suffered ACL injury and their healthy counterparts. The results revealed no statistically significant differences between the groups in any force–time metrics of interest within both the eccentric and concentric phases of the CMJ (e.g., concentric peak force, countermovement depth, eccentric mean power). In addition, no significant differences have been found in inter-limb asymmetry measures (i.e., peak takeoff and landing force).

Previously published research reports have found notable differences in various kinetic and kinematic parameters before and after ACLR [32,33,34], as well as between injured and healthy athletes [10,13,17], and operated vs. non-operated limbs [35,36,37], which is contradictory to the results obtained in the present study. Also, previous research had found that inter-limb deficiencies in force production tend to remain present 6–9 months post-ACLR [38,39,40] and appear to normalize within 2 years [12]. Hence, the possible reason for the discrepancy in the aforementioned findings may be attributed to the time point at which the athletes were assessed (i.e., 6–9 months vs. 11–13 months post-ACLR). For example, when studying 33 non-professional athletes, Legani et al. [40] found that knee functional performance was unsatisfactory in most patients 6 months post-ACLR. However, when the same cohort of participants was reassessed 12 months post-ACLR, many of them showed performance improvements and met the return to sport criteria [40]. Similarly, Renner et al. [41] have observed a gradual increase in peak vertical ground reaction force across multiple testing sessions within a 3–6-month timeframe. Thus, we can assume that the athletes examined in the present study already passed the critical time point at which notable differences in force–time metrics within both the eccentric and concentric phases of the CMJ could be observed. Moreover, these findings may imply the effectiveness of the rehabilitative procedures implemented by the medical staff as well as the athletes’ willingness to adhere to them, which has shown to be one of the key elements for successful recovery and return to sport [42]. 

Another possible reason for the discrepancy in the findings between the previously published research reports and the results obtained in the present investigation pertains to the testing methodology (i.e., CMJ vs. drop jump). Drop jump has been more frequently used for inter-limb asymmetry and post-ACLR performance assessment than the CMJ [10,37,43]. While both movements require eccentric contraction followed by a rapid concentric contraction, the drop jump requires a notably greater eccentric muscle involvement, as it entails more intense landing alongside shorter ground contact times [10,44]. For example, Paterno et al. [36] found greater ground reaction forces and loading rates in the healthy limb when compared to the operated limb during a drop jump test performed approximately 27 months post-ACLR. Similarly, in a recently published study, Ohji et al. [45] found significantly lower RSI and limb symmetry index within a group of patients that did not satisfy return to sport criteria than the one that did, when performing single-leg drop jumps approximately 13 months post-ACLR. This is particularly important to note, as RSI has been used as one of the key performance indicators for a successful return to sport [45]. While further research is warranted on this topic, we assume that the CMJ may not present a sufficient neuromuscular performance stimulus, such as a drop jump, that would expose lower-limb asymmetries and strength and power deficiencies 11–13 months post-ACLR. This assumption can be further supported by the results obtained by Bobbert et al. [46], who found that both moment and mean power output at the knee and ankle joints, including electromyography of gastrocnemius muscle, were significantly lower in CMJ than drop jump. 

Nonetheless, while drop jump may present itself as a better tool for assessing lower-limb asymmetries and strength and power deficiencies post-ACLR, research has shown that this test demonstrated poor sensitivity and specificity when used as a screening tool for predicting ACL injury [19,47]. Specifically, Krosshaug et al. [19] examined a large cohort of 710 athletes (i.e., handball players) that performed drop jumps as a part of their comprehensive pre-season performance screening and found that knee valgus angle, knee abduction moment, vertical ground reaction force, and knee flexion angle were not associated with an elevated ACL injury risk. Similar findings were obtained by Petushek et al. [47], who found that neither single-leg squat nor drop jump were capable of distinguishing athletes who sustained an ACL injury vs. the ones who did not (i.e., 429 handball and 451 soccer players). On the other hand, Ruffineux et al. [11] studied non-professional volleyball players and found that a six-week training protocol that implemented CMJ was more effective in eliciting improvements in jump height than one that implemented drop jump instead of CMJ. So, based on these findings, we can conclude that both CMJ and drop jump testing modalities are valuable, including their single-leg variations. Despite no differences in CMJ performance being observed in this study, to obtain a comprehensive insight into athletes’ neuromuscular performance capabilities, it seems that these two tests should supplement rather than substitute each other. Moreover, test selection (i.e., CMJ vs. drop jump) may be based on the circumstances that pertain to each athlete (i.e., ACL injury risk screening vs. post-ACLR).

Lastly, it is interesting to note that no differences in any of the force–time metrics of interest were present between healthy and ACL-injured athletes in both male and female cohorts of soccer players. Besides the fact that ACL injury occurrence is considerably greater in female than male athletes [8,48], the return to sport timeline seems to be similar between the genders [7]. Still, female athletes tend to show greater quadriceps strength deficits post-ACLR compared to males [49]. This seems to be contradictory to the findings of the present study, as no significant differences were observed in mean and peak force and power production within both eccentric and concentric phases of the CMJ. As previously indicated, this can be attributed to the quality of the rehabilitation program and athletes’ adherence to it, which lasted at least 9 months and was performed in the same clinic by the same team of medical professionals. Also, this nine-month timeframe was found to be of critical importance in previously published research [38,50]. For example, an early return to sport (i.e., <9 months) was associated with a seven-fold increased rate of sustaining a second ACL injury, likely due to [50]. Moreover, Thomson et al. [38] found a presence of asymmetries in vertical force production during running in male soccer players that progressed with an increase in running speed when athletes returned to the sport in less than 9 months post-ACLR, despite meeting the criteria-based rehabilitation program. However, the same group of authors observed a reversed trend in healthy age-matched individuals as well as athletes who returned to sport >9 months post-ACLR [38], which corresponds to the rehabilitation timeframe that the athletes examined in this study were exposed to. 

While providing a deeper insight into lower-body neuromuscular performance differences between ACL-injured athletes and their healthy counterparts, this study is not without limitations. The lack of pre-injury data for each athlete (e.g., baseline) is one of the main limitations of this investigation, as this type of data could provide detailed pre–post performance assessment on a within-subject basis. Also, the cohort of participants was homogenous, as it included only semi-professional soccer athletes competing within the same level of play. Therefore, further research is warranted to examine if these findings remain applicable to other competitive levels (e.g., collegiate, professional), as well as other team and individual sports (e.g., tennis, basketball). 

In conclusion, the results of the present study revealed no statistically significant differences in CMJ force–time metrics (e.g., concentric peak force, eccentric mean power) and inter-limb asymmetry measures (i.e., peak takeoff and landing force) between ACL-injured and healthy non-injured semi-professional male and female soccer players. Thus, besides implying the effectiveness of the nine-month recovery protocol, these findings suggest that the CMJ may not present a sufficient neuromuscular performance stimulus needed to expose lower-limb asymmetries and strength and power deficiencies 11–13 months post-ACLR.

## Figures and Tables

**Table 1:** Descriptive statistics, mean and standard deviations (x̄ ± SD) or median and interquartile range (M[IQR]), for each dependent variable, including a between-group statistical comparison.

Variable [Unit]	Male			Female		
	Healthy	ACL	*p*-Value	Healthy	ACL	*p*-Value
Eccentric phase						
ECC duration [s]	0.59 ± 0.14	0.59 ± 0.12	0.915	0.62 ± 0.10	0.63 ± 0.08	0.849
ECC velocity [m/s]	−1.09 ± 0.29	−1.13 ± 0.38	0.672	−0.84 ± 0.13	−0.91 ± 0.18	0.261
ECC peak force [N/kg]	20.5 ± 3.1	22.1 ± 3.5	0.126	19.0 ± 2.6	18.7 ± 2.7	0.691
ECC mean force [N/kg]	9.8 [0.1]	9.9 [0.3]	0.499 *	9.8 ± 0.1	9.9 ± 0.1	0.694
ECC peak power [W/kg]	13.7 ± 4.7	15.6 ± 8.0	0.448	9.9 ± 2.8	8.9 ± 2.2	0.312
ECC mean power [W/kg]	5.4 ± 1.3	5.9 ± 1.8	0.220	4.7 ± 0.7	4.8 ± 0.7	0.602
Concentric phase						
CON duration [s]	0.30 ± 0.07	0.29 ± 0.05	0.452	0.29 ± 0.04	0.30 ± 0.06	0.679
CON velocity [m/s]	2.8 [0.3]	2.9 [0.4]	0.920 *	2.3 ± 0.2	2.4 ± 0.2	0.151
CON peak force [N/kg]	24.5 ± 2.2	24.1 ± 2.5	0.585	20.7 ± 1.4	22.0 ± 2.1	0.098
CON mean force [N/kg]	19.1 ± 1.7	19.5 ± 1.9	0.551	17.2 ± 0.9	17.7 ± 1.7	0.422
CON peak power [W/kg]	56.6 ± 8.4	53.8 ± 7.3	0.303	39.9 ± 5.0	44.4 ± 5.9	0.510
CON mean power [W/kg]	26.9 ± 4.3	28.8 ± 4.4	0.212	21.0 ± 3.0	21.4 ± 0.4	0.811
Asymmetry						
Takeoff force [%]	3.4 [5.6]	6.0 [8.7]	0.184 *	6.2 ± 3.6	6.1 ± 3.9	0.914
Landing force [%]	6.8 [12.7]	11.8 [15.7]	0.101 *	18.4 ± 14.4	17.8 ± 11.8	0.902
Other						
Contraction time [s]	0.89 ± 0.17	0.88 ± 0.15	0.750	0.92 ± 0.12	0.93 ± 0.13	0.820
Jump height [cm]	36.1 [9.9]	37.8 [13.1]	0.960 *	23.2 [8.6]	27.7 [5.4]	0.190 *
RSI-modified [ratio]	0.46 [0.18]	0.43 [0.25]	0.764 *	0.28 ± 0.1	0.32 ± 0.1	0.146
Countermovement depth [cm]	−34.3 ± 10.6	−34.8 ± 6.9	0.851	−26.6 [7.6]	−28.5 [7.2]	0.938 *

Note: CON—concentric; ECC—eccentric; ACL—anterior cruciate ligament; RSI—reactive strength index (modified); (*) variables that violated the normality assumption expressed as median (interquartile range).

## Data Availability

The data are not publicly available due to IRB-imposed restrictions.
